# Oxide Thin-Film Transistor-Based Vertically Stacked Complementary Inverter for Logic and Photo-Sensor Operations

**DOI:** 10.3390/ma12233815

**Published:** 2019-11-20

**Authors:** Hyo-Jun Joo, Min-Gyu Shin, Hwan-Seok Jung, Hyun-Seok Cha, Donguk Nam, Hyuck-In Kwon

**Affiliations:** 1 School of Electrical and Electronics Engineering, Chung-Ang University, Seoul 06974, Korea; hyojunjun@naver.com (H.-J.J.); 18alsrb@naver.com (M.-G.S.); hwanseok518@cau.ac.kr (H.-S.J.); ckgustjr0803@naver.com (H.-S.C.); 2 School of Electrical and Electronic Engineering, Nanyang Technological University, 50 Nanyang Avenue, Singapore 639798, Singapore; dnam@ntu.edu.sg

**Keywords:** oxide TFT, vertically stacked complementary logic inverter, SnO, IGZO, photo-sensor

## Abstract

Numerous studies have addressed the utilization of oxide thin-film transistor (TFT)-based complementary logic circuits that are based on two-dimensional (2D) planar structures. However, there are fundamental limits to the 2D planar structured complementary logic circuits, such as a large dimension and a large parasitic resistance. This work demonstrated a vertically stacked three-dimensional complementary inverter composed of a p-channel tin monoxide (SnO) TFT and an n-channel indium-gallium-zinc oxide (IGZO) TFT. A bottom-gate p-channel SnO TFT was formed on the top-gate n-channel IGZO TFT with a shared common gate electrode. The fabricated vertically stacked complementary inverter exhibited full swing characteristics with a voltage gain of ~33.6, a high noise margin of 3.13 V, and a low noise margin of 3.16 V at a supplied voltage of 10 V. The achieved voltage gain of the fabricated complementary inverter was higher than that of the vertically stacked complementary inverters composed of other oxide TFTs in previous works. In addition, we showed that the vertically stacked complementary inverter exhibited excellent visible-light photoresponse. This indicates that the oxide TFT-based vertically stacked complementary inverter can be used as a sensitive photo-sensor operating in the visible spectral range with the voltage read-out scheme.

## 1. Introduction

Oxide-semiconductor-based thin-film transistors (TFTs) are promising devices for the implementation of electronic circuits in large areas owing to their attractive properties such as high carrier mobility, high uniformity, and low-temperature process compatibility [1,2,3,4,5]. So far, various studies have demonstrated complementary logic circuits using oxide TFTs because they have several advantages in comparison to n-type logic circuits, such as low power consumption, high voltage gain, and good noise immunity [6,7,8,9,10,11,12,13,14,15]. While most oxide TFT-based complementary logic circuits reported thus far were based on two-dimensional (2D) planar structures, there exist fundamental limits to the 2D planar structured complementary logic circuits, such as a large dimension due to the horizontally distributed n- and p-channel transistors and a large parasitic resistance caused by long interconnection lengths. The three-dimensional (3D) stacked structure requires more difficult processing techniques in comparison to the 2D planar structure including thermal budget optimization. However, the 3D stacked structure has recently attracted much attention since it can significantly increase the packing density and also improve the drivability of microelectronic circuits by reducing the interconnection lengths and parasitic resistances [16,17]. In addition, Lin et al. [18] showed that the obtained parasitic capacitance of the 3D stacked silicon complementary inverter was also lower than that of the 2D planar structure. The experimental realization of 3D stacked structure remains challenging, and therefore, only a few research groups have reported vertically stacked complementary inverters composed of oxide TFTs or oxide/organic TFTs [14,15,16,17,19,20]. Particularly, to the best of our knowledge, there exists no experimental demonstration of the 3D stacked complementary inverter using indium-gallium-zinc oxide (IGZO) TFT and tin monoxide (SnO) TFT, that are the most representative n-channel and p-channel oxide TFTs.

In this work, we demonstrated a 3D stacked complementary inverter composed of an n-channel IGZO and a p-channel SnO TFT for the first time. The fabricated 3D stacked complementary inverter exhibited full swing characteristics with a high voltage gain of ~33.6 at a supplied voltage (*V*_DD_) of 10 V. In addition, we investigated the photoresponse of the 3D stacked complementary inverter in the visible region. The obtained results show that the fabricated inverter can be used as a sensitive visible light sensor using the voltage read-out scheme.

## 2. Experimental Procedure

**Figure 1:**
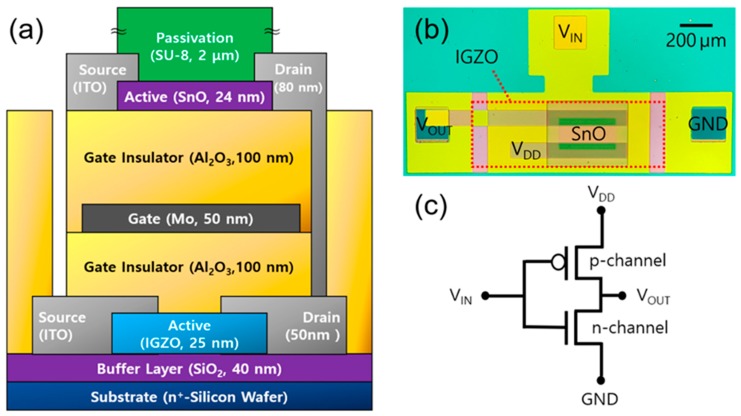
(**a**) schematic cross-sectional view, (**b**) optical image, and (**c**) circuit diagram of the vertically stacked complementary logic inverter composed of p-type tin monoxide (SnO) and n-type indium-gallium-zinc oxide (IGZO) thin-film transistors (TFTs).

Figure 1a shows the schematic cross-sectional view of the fabricated vertically stacked complementary logic inverter. A 25-nm-thick IGZO channel layer was deposited by RF magnetron sputtering without intentional substrate heating using a 4-inch IGZO target (In:Ga:Zn:O = 1:1:1:4 mol%, purity: 99.99 %) on a heavily doped n-type Si wafer covered by a 40-nm-thick thermal SiO_2_ and patterned by a lift-off process. During the deposition of the IGZO channel layer, the working pressure, Ar flow rate, and sputtering power were 5.0 mTorr, 20 sccm, and 50 W, respectively. A 50 nm-thick ITO layer was deposited by DC sputtering as the source/drain electrodes and was patterned using a lift-off process. Then, the deposited thin films were subjected to simultaneous ultraviolet and thermal (SUT) treatments at 150 °C for 1 h in air using a hot plate and ultraviolet light. Ultraviolet light irradiation was performed using a mercury-sourced ultraviolet light with wavelengths of 185 nm (at 10%) and 254 nm (at 90%) during the SUT treatment. The photon flux density of the ultraviolet light was fixed at 15 mW/cm^2^. The SUT treatments were reported to be effective in the densification and condensation of various sputter and solution-processed metal oxide thin films and improve the electrical characteristics and stabilities of IGZO TFTs [21,22,23,24]. A 100-nm-thick Al_2_O_3_ film was deposited at 200 °C to serve as the gate insulator of the IGZO TFT by atomic layer deposition (ALD) using Al(CH_3_)_3_ (trimethylaluminum (TMA)) and H_2_O as precursors. The Al_2_O_3_ was patterned using photolithography and wet etching to expose the source/drain contact holes. A sputtered molybdenum film with a thickness of 50 nm was used as the common gate electrode and patterned by a lift-off process.

Another 100 nm-thick Al_2_O_3_ film was deposited at 200 °C by ALD on top of the IGZO TFT to serve as the gate insulator for the SnO TFT. The gate insulator for the SnO TFT was patterned using photolithography and wet etching to expose the common gate and source/drain contact holes of the IGZO TFT. Next, a 24 nm-thick SnO layer was deposited using the RF magnetron sputtering method without intentional substrate heating using a 3-inch Sn target and patterned by a lift-off process. Then, it was annealed at 180 °C for 30 min using hot a plate. During the deposition of the SnO channel layer, the working pressure, gas mixing ratio (Ar/O_2_), and sputtering power were 3.0 mTorr, 90/4 (sccm/sccm), and 50 W, respectively. An 80 nm-thick ITO layer was deposited by DC sputtering as the source/drain electrodes of the SnO TFT and was patterned using a lift-off process. Next, a 2 μm-thick SU-8 layer was formed using the spin coating process as a passivation layer following the formation condition described in detail in our previous work [25]. Finally, the sample was post-annealed at 180 °C for 15 min in air. The optical image and circuit diagram of the fabricated vertically stacked complementary inverter are illustrated in Figure 1b,c, respectively. All electrical measurements for the TFTs and circuits were conducted at room temperature in air using an Agilent 4156C precision semiconductor parameter analyzer (Agilent Technologies., Santa Clara, CA, USA).

## 3. Results and Discussion

### 3.1. Thin Film Characterization

**Figure 2:**
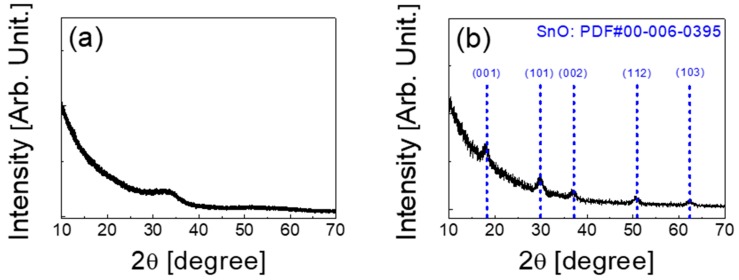
XRD patterns of the (**a**) IGZO and (**b**) SnO thin films used as channel layers in this work.

Figure 2a,b display the X-ray diffraction (XRD, Rigaku, Tokyo, Japan) patterns obtained from the IGZO and SnO thin films deposited on the SiO_2_/Si substrate, respectively. The thickness of the thin-films were 25 nm for the IGZO film and 24 nm for the SnO film, respectively. The crystal structures of the IGZO and SnO thin-films were characterized by a D8 Advance X-ray diffractometer from Bruker AXS using *CuK*_α1_ radiation (λ = 1.54056 Å). As shown in Figure 2a, the observed broad peak in the range of 30° to 35° is considered as an amorphous halo peak that comes from the IGZO film [26]. Figure 2b presents the crystalline status of the SnO thin-film. SnO possesses a tetragonal PbO-type (P4/nmm) crystallographic structure, in which the spherical Sn 5 s orbitals and O 2p orbitals have almost the same energy levels for effective interaction. Therefore, they can form delocalized and isotropic hybridized orbitals that provide an effective hole transport path [27,28]. Owing to its potential for high hole mobilities, SnO has attracted special attention as a promising p-type oxide semiconductor.

The XRD patterns in Figure 2b are well matched with the (001), (101), (002), (112), and (103) planes of the tetragonal SnO phase (Powder Diffraction File (PDF) card No. 00-006-0395). The average grain size (D) calculated from the SnO (101) peak was 126 Å. The grain size was calculated based on the Debye-Scherrer equation [29]:

(1) $$D=\frac{0.9\lambda }{\beta \cos\theta }$$

where λ is the X-ray wavelength, *β* is the full width at half maximum of the diffraction peak, and* θ* is the Bragg angle, respectively.

**Figure 3:**
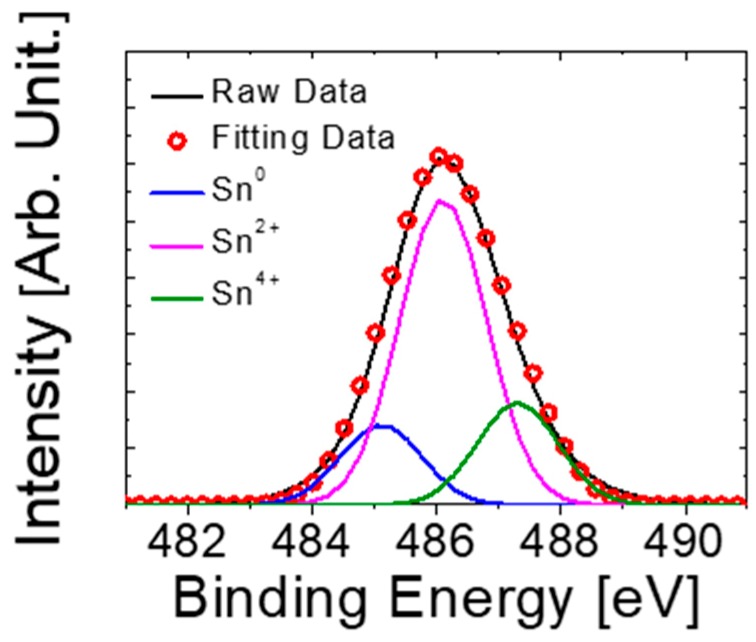
X-ray photoelectron spectroscopy (XPS) Sn 3*d*_5/2_ spectra of the tin oxide thin-film deposited on the SiO_2_/Si substrate.

Figure 3 shows the X-ray photoelectron spectroscopy (XPS, Thermo Fisher Scientific, East Grinstead, UK) Sn 3*d*_5/2_ spectra of the tin oxide thin-film deposited on SiO_2_/Si substrate. The Sn 3*d*_5/2_ spectra are deconvoluted into three sub-peaks, corresponding to Sn^0^, Sn^2+^, and Sn^4+^ components. The binding energy values are fixed at 485.1, 486.0, and 487.3 eV, respectively [30]. The XPS results showed that the deposited thin-film was composed of Sn, SnO, and SnO_2_, but the p-type SnO/Sn^2+^ was the dominant phase as observed in Figure 2b.

### 3.2. Electrical Characteristics of N-Channel IGZO and P-Channel SnO TFTs

**Figure 4:**
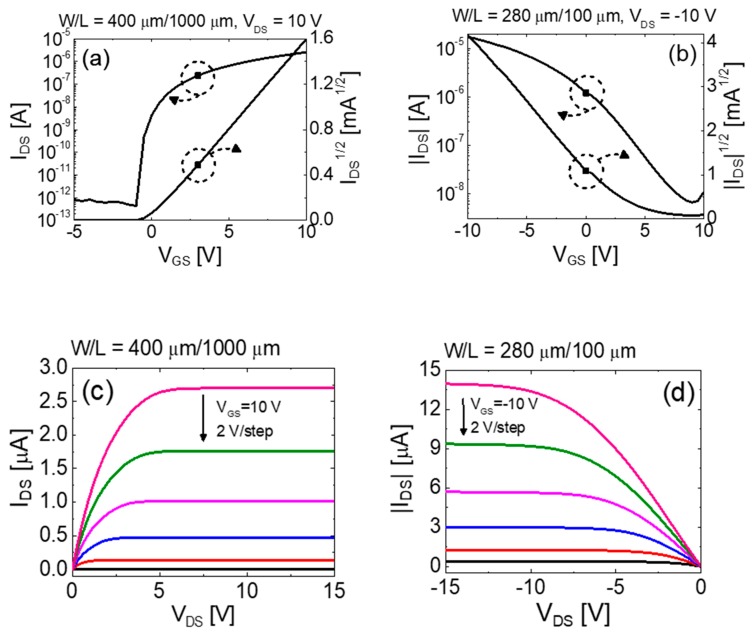
(**a**,**b**) transfer and (**c**,**d**) output curves of the fabricated vertically stacked top-gate IGZO and bottom-gate SnO TFTs, respectively.

Because it is essential to match the characteristics of n- and p-channel TFTs to obtain excellent complementary logic circuits, individual current-voltage characteristics of vertically stacked top-gate IGZO and bottom-gate SnO TFTs were investigated. The channel width (*W*) and length (*L*) of the devices were *W*_n_ = 400 μm and *L*_n_ = 1000 μm for the n-channel IGZO TFT and *W*_p_ = 280 μm and *L*_p_ = 100 μm for the p-channel SnO TFT, respectively. The transfer curves (drain current *I*_DS_ versus gate-source voltage *V*_GS_) and output curves (*I*_DS_ versus drain-source voltage *V*_DS_) are shown in Figure 4a,c and Figure 4b,d for n-channel and p-channel TFTs, respectively. The transfer curves were measured at *V*_DS_ = 10 V for IGZO TFT and *V*_DS_ = −10 V for SnO TFT. The values of saturation mobility (*μ*_SAT_), on/off current ratio (*I*_ON_/*I*_OFF_), threshold voltage (*V*_TH_), and subthreshold swing (*SS*) were 7.61 cm^2^/V⋅s, 4.30 × 10^8^, 0.03 V, and 0.29 V/dec for IGZO TFT and 1.34 cm^2^/V⋅s, 5.75 × 10^3^, 3.15 V, and 3.62 V/dec for SnO TFT, respectively. Here, the values of *μ*_SAT_ were calculated from the following equation:

(2) $$\mu_{\mathrm{SAT}}=\frac{2L}{WC_{i}}{\left(\frac{\partial \sqrt{I_{DS}}}{\partial V_{GS}}\right)}^{2}$$

where *C_i_* represents the capacitance of the gate insulator per unit area. The *μ*_SAT_ values of each TFT are comparable with those of previous reports, thereby demonstrating that the stacking process did not deteriorate their performances [31,32]. The values of *V*_TH_ were extracted by extrapolating a straight line in (*I_DS_*)^1/2^-*V_GS_* plot on the *V_GS_* axis. The *SS* values which indicate the necessary *V_GS_* to increase *I_DS_* by one decade were calculated from the inverse of the maximum slope of the transfer characteristics. Typical output curves of the IGZO and SnO TFTs are shown in Figure 4c,d, respectively. It is evident that the output curves exhibit clear pinch-off and current saturation.

### 3.3. Static Performance of Vertically Stacked Complementary Inverter

**Figure 5:**
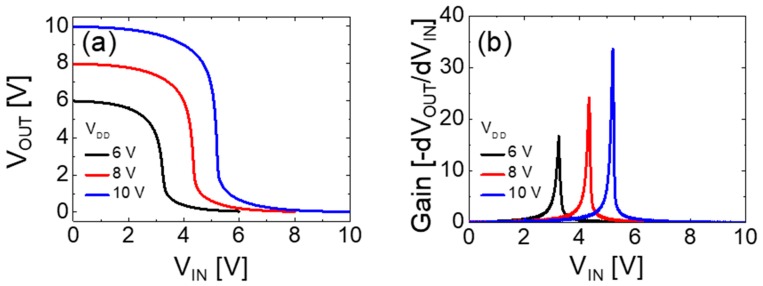
(**a**) voltage transfer characteristics and (**b**) static DC gain of the vertically stacked complementary inverter with p-channel SnO TFT (*W*_n_/*L*_n_ = 400 μm/1000 μm) fabricated on top of the n-channel IGZO TFT (*W*_p_/*L*_p_ = 280 μm/100 μm) at different *V*_DD_s of 6, 8, and 10 V.

The static performance of the vertically stacked complementary inverter was evaluated using its switching threshold voltage (*V*_M_), static circuit gain (*A*_V_ = |*dV*_OUT_/*dV*_IN_|), and noise margin values extracted from the measured inverter voltage transfer characteristics (VTCs). Figure 5a,b show the VTC and static DC gain values of the vertically stacked complementary inverter at different *V*_DD_s of 6, 8, and 10 V, respectively. The values of *W*_n_/*L*_n_ and *W*_p_/*L*_p_ of the IGZO and SnO TFTs were 400 μm/1000 μm and 280 μm/100 μm, respectively. The large value of *W*/*L* ratio of the SnO TFT compare to that of the IGZO TFT was employed to compensate the difference in *μ*_SAT_ values of IGZO and SnO TFTs and to optimize the inverter performance. Clear inverter action with a full swing is observed in Figure 5a. The performance of the fabricated complementary inverter at different *V*_DD_s is summarized in Table 1.

As shown in Figure 5b, the voltage gain increases with the *V*_DD_ and reaches ~33.6 at *V*_DD_ = 10 V, which is higher than that of the oxide TFT-based vertically stacked complementary inverters reported in previous works at the same *V*_DD_ [14,15]. The relatively high voltage gain may be attributed to the high output resistance of both TFTs, as shown in Figure 4. It is expected that the gain of the inverter can be more increased by improving the mobility and output resistance of TFTs [33]. The noise margin values were extracted from the static VTC shown in Figure 5a. The input-high voltage (*V*_IH_) and input-low voltage (*V*_IL_) are the input voltage values where the slope of VTC is equal to −1. The output-high voltage (*V*_OH_) and output-low voltage (*V*_OL_) are the output voltage values for the input-low voltage and input-high voltage, respectively. For *V*_DD_ = 10 V, the noise margin high (*N*_MH_ = *V*_OH_ − *V*_IH_) and noise margin low (*N*_ML_ = *V*_IL_ − *V*_OL_) were 3.13 and 3.16 V, respectively. The balanced noise margin may be attributed to the well optimized geometric aspect ratio between the pull-up SnO and pull-down IGZO TFTs.

### 3.4. Visible Light Photoresponse of Vertically Stacked Complementary Inverter for Optoelectronic Applications

**Figure 6:**
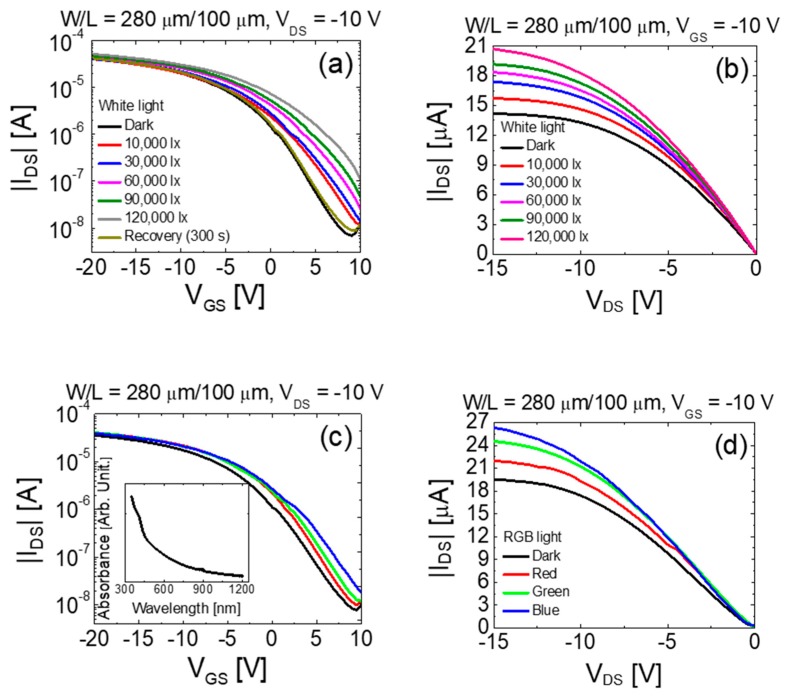
(**a**) transfer and (**b**) output curves of the vertically stacked bottom-gate SnO TFTs under white light illumination with different intensities. (**c**) transfer and (**d**) output curves of the SnO TFT under red-green-blue (RGB) light illumination. The RGB light intensity is fixed at ~6.5 mW/cm^2^. The inset of (**c**) shows the UV-vis absorption spectra of the deposited SnO thin-film.

To maximize the potential of vertically stacked complementary inverters beyond the logic applications by including optoelectronic applications such as photo-sensors, we investigated their photoresponse. First, the characteristics of the vertically stacked bottom-gate SnO TFT device upon illumination with visible light was investigated. We performed the electrical measurements in the dark as well as under light illumination at different light intensities and wavelengths. The effects of light illumination on top-gate IGZO TFT was not significant in the turn-on region because the IGZO film is covered by the Mo gate electrode. Figure 6 shows the transfer and output curves of the vertically stacked bottom-gate SnO TFT in the dark and when illuminated on the top of the device with white (wavelength ~ 420–780 nm), red (wavelength ~ 625–630 nm), green (wavelength ~ 505–535 nm), and blue (wavelength ~ 460–465 nm) light-emitting-diodes (LEDs). The SU-8 for the passivation layer is an optically transparent material for the light with a wavelength of above 400 nm [34], which implies that almost all photons from the LEDs pass through the SU-8 passivation layer without being absorbed.

Figure 6a,b show the transfer and output curves of the SnO TFT obtained under white light illumination with different intensities, respectively. It is evident from Figure 6a that the transfer curve shifts in the positive direction with an increase in *SS*, and *I*_OFF_ under light illumination. This phenomenon can be attributed to the increase in the conductivity in SnO because the absorbed photon can increase the photogeneration in holes and electrons under light illumination [35]. More photons are expected to be absorbed under more intense light illumination. The transfer curve in Figure 6a shows the recovery behavior in dark condition, following a light illumination of 120,000 lx. The transfer curve recovered to the initial state within 300 s after turning-off a light source. The mechanism which can explain the recovery behavior in Figure 6a is the trapping and detrapping of electrons in bulk defect states of SnO thin-film. The photo generated electrons can be trapped in the bulk states, while holes are able to be more mobile in the semiconductor and they are likely to be swept away. A thermal release of trapped electrons causes the recovery behavior of the transfer curve after turning-off the light. Our previous experiment described the recovery behavior of SnO TFT after terminating the light illumination in detail [36]. The photogenerated carrier effect is also observed in the output characteristics, as shown in Figure 6b. The output resistance decreased with an increase in the intensity of the white light. Figure 6c,d show the transfer and output curves in the dark and under red-green-blue (RGB) light illumination for a fixed intensity of ~6.5 mW/cm^2^. Figure 6c shows that the transfer curve shifts in the positive direction with an increase in *SS* and *I*_OFF_ under RGB light illumination as compared to the dark condition. The characteristics of the device further changed as the wavelength decreased from the red to the blue light. The output resistance also further decreased with a decrease in the wavelength of the light, as shown in Figure 6d. These results suggest that the changes in the electrical properties of SnO TFT under light illumination strongly depend on the intensity and wavelength (photon energy) of the illuminated light. The inset of the Figure 6c depicts the absorption spectra of the deposited SnO thin-film within the wavelength range of 350–1200 nm. The absorbance increased with a decrease of the wavelength. This result is consistent with the results shown in Figure 6c,d.

**Figure 7:**
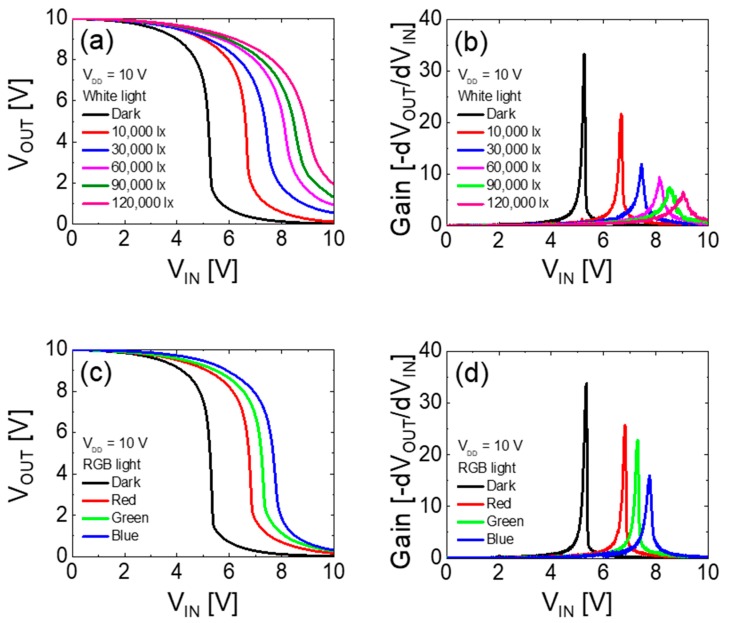
(**a**) voltage transfer characteristic (VTC) and (**b**) static voltage gain curves of the vertically stacked complementary inverter under white light illumination with different intensities. (**c**) VTC and (**d**) static voltage gain curves of the inverter under RGB light illumination. The VTCs and corresponding voltage gain curves were measured at *V*_DD_ = 10 V. The fixed RGB light intensity was ~6.5 mW/cm^2^.

The effects of incident light on the electrical characteristics of the vertically stacked complementary inverter were studied by measuring the photoresponse of the inverter circuit with respect to the illumination intensity and wavelength of the light. The vertically stacked complementary inverter showed excellent photoresponse under visible light illumination. Figure 7a,b show the VTC and static DC gains obtained as a function of the intensities of white light. The *V*_M_ was shifted from 5.19 to 8.92 V and the static gain of the inverter was decreased from ~33.2 to ~6.5 with an increase in the light intensity from 0 to 120,000 lx at *V*_DD_ = 10 V.

Similar photoresponse was observed under RGB light illumination condition, where the initial VTC curve moved toward a more positive direction as the wavelength of light decreased, as shown in Figure 7c. The *V*_M_ was 5.26 V (in dark) and increased under illumination to 6.75 V (red light), 7.22 V (green light), and 7.67 V (blue light) at *V*_DD_ = 10 V. The values of static voltage gain of the inverter were ~33.5 (in dark), ~25.7 (red light), ~22.8 (green light), and ~16.1 (blue light) at *V*_DD_ = 10 V, as shown in Figure 7d. The positive shift in *V*_M_ occurred owing to the positive shift in *V*_TH_ of the SnO TFT by photogenerated charge carriers under illumination. The decrease in the voltage gain under light illumination can be attributed to the decrease in output resistance of the SnO TFT, as shown in Figure 6 [33,37,38]. The obtained results show the potential for a vertically stacked complementary inverter to be used in sensitive optoelectronic devices that can detect a specific wavelength within the visible light. 

## 4. Conclusions

In summary, we demonstrated a vertically stacked complementary inverter using n-channel IGZO and p-channel SnO TFTs. A bottom-gate p-channel SnO TFT was stacked on top of a top-gate n-channel IGZO TFT using a shared common gate electrode to implement this geometry. The fabricated inverter exhibited clear inverter action with full swing characteristics. The static voltage gain increased with an increase in *V*_DD_ and reached ~33.6 when *V*_DD_ was equal to 10 V. We also investigated the performance of the vertically stacked complementary inverter under light illumination. The results showed that the fabricated vertically stacked complementary inverter exhibited excellent visible-light photoresponse; this implied that the fabricated complementary inverter can be applied in optoelectronic applications such as photo-sensors and imaging systems.

## Figures and Tables

**Table 1:** Performance of the vertically stacked complementary inverter at different *V*_DD_s.

*V*_DD_ [V]	*V*_M_ [V]	*A*_V_ [V]	*N*_ML_ [V]	*N*_MH_ [V]
6	3.15	16.7	1.72	1.70
8	4.26	24.1	2.55	2.30
10	5.18	33.6	3.16	3.13
